# Remarks on the Pathology of Phlegmasia Doleus

**Published:** 1856-01

**Authors:** Hayden Coe

**Affiliations:** Atlanta, Ga.


ATLANTA

Medical and Surgical Journal.

Vol. I.] JANUARY, 1856. [No. 5.

### ORIGINAL COMMUNICATIONS.

### Article I.—*Remarks on the Pathology of Phlegmasia Doleus.* By Hayden Coe, M. D., Atlanta, Ga.

Although this disease has long been known to the profession, yet the great variety of opinions as to the real nature of this disease, which have been given by different authors from time to time, is a conclusive argument, or goes far, at least, to show that we may not yet well understand its true nature. It is not necessary for me to rehearse the numerous theories which have been advanced by Mauriceau, Pugos, White, Trye, Hull and others—most of which have long since been abandoned—they are too familiar to the profession to need repitition here. One only shall we notice—and the only one which has received much attention from the profession of late—is that advanced by Dr. Davis, afterwards supported by Dr. Robert Lee and others, viz: that Phlegmasia Dolens is Crural Phlebitis. But many of the symptoms which occur in Phlegmasia Dolens are so entirely dissimilar from those which occur in Phlebitis, that this theory also is not fully satisfactory, and there are some Pathologists at the present time, we believe, who are still averse (and we think with reason) to asscribing all the phenomena which occur in Phlegmasia Dolens to inflammation of the veins. We have always found very great difficulty in reconciling the symptoms, which occur in Phlegmasia Dolens with those which occur in Phlebitis. That Dr. Davis and Dr. Lee state what they saw in dissections is so, beyond a doubt, but that their cases were all true cases of Phlegmasia Dolens, we think admits of some doubt, although it may be possible that pus may be found in the veins of some aggravated cases of this disease, especially such as prove fatal; but if so, we must look upon it as a secondary effect, and does not take place until the disease is far advanced. But in the majority of cases, a very large number of which recover, a disease attended with so little danger—probably not one case in a hundred proves fatal—I have never seen a fatal case—can have but little connection with so formidable a disease as Phlebitis, in which more than one-half the cases prove fatal under the most efficient course of treatment.

The type of fever in Phlebitis "is typhoid, putrid or adynamic. Indeed, the term putrid," says Bouellaud, "is perfectly applicable, since after, nay even before death, there are unequivocal signs of decomposition." Ribes says, "Phlebitis is a serious malady, and is often quickly mortal."

The swelling progresses much slower in Phlebitis than in Phlegmasia Dolens, and when inflammation exists in the superficial veins, redness is seen along the course of the vessels, but is never seen in Phlegmasia Dolens. In Phlegmasia Dolens the type of the fever is remittent or intermittent, although this disease occurs in lying-in women, when we would most expect a continued form of fever. The swelling is rapid, extending over the whole limb in a few hours, the limb being whiter than natural, also great increase of sensibility, with loss of motion, showing great derangement of the nervous tissue.

But it is not so much our purpose to show that the pathology of this disease is not Phlebitis, as it is to show that it is a reflex disease—its primary seat of irritation being in the uterus, or its appendages—which irritation being conveyed through the medium of the nerves to the lumbar and sacral ganglia, one or both, producing permanent irritation in those ganglia, which affects the efferent nerves arising from those ganglia, and is felt at the periphery of those nerves. If the spinal column is composed of separate ganglia, connected with each other only by fibrous commissures and reflexed action carried on through those ganglia, independent of the cerebrum and irritation, or stimuli, applied to the periphery of the efferent nerves, produce similar action through the medium of these ganglia, as if applied directly to the ganglia, or the afferent nerves, as laid down by physilogists, and proved by experiment to be true, it appears equally true, that irritation long continued in the uterus, may produce permanent irritation in the lumbar and sacral ganglia, especially when we consider the intimate connection, which is as follows: The uterus is supplied with nerves derived from the hypogastric, spermatic and sacral plexus. The external branches from the lumbar gruglia communicate with the lumbar nerves, the internal branches pass, some to form the hypogastric plexus, others the aortic plexus, which also sends branches to the hypogastric plexus. The hypogastric plexus is formed by the termination of the aortic plexus, and by the union of branches from the lower lumbar gruglia, the external branches from the sacral gruglia communicate with the sacral and congeal nerves, while the internal branches communicate freely with the lateral divisions of the hypogastric plexus and are distributed to the pelvic visera. It looks very reasonable to us, that irritation in the uterus, applied to these nerves, might be readily communicated to the spinal column, producing irritation in the lumbar and sacral portion, thereby affecting all parts to which the nerves are distributed, arising from the diseased portion of the spinal column. It appears to us, that it is owing to the intimate and extensive connection of the hypogastric plexus with others, that causes the stomach, the liver, the lungs, and almost all other of the abdominal and thorasic viscera to become affected in uterine disease.

We shall now enumerate some of the symptoms as given by different authors, and see how far they can be reconciled with the theory as above stated.

1st. Churchill says: "This disease usually occurs in women who have suffered from uterine irritation, or inflammation, and may even be caused by such condition of the uterus. The ordinary premonitory symptoms commence with pain and uneasiness in the lower part of the abdomen, extending along the brim of the pelvis; the patient is irritable, depressed, complains of great weakness."

The above symptoms indicate nothing more than uterine irritation of some kind existing, and an irritable and depressed state of the nervous system.

Churchill further says: "Sometimes, however, there are no precursory symptoms, the patient being suddenly seized with pain in the calf of the leg; or it may commence like rheumatism, affecting the back and hip joint."

How is it that Crural Phlebitis can produce such sudden and violent pain in the calf of the leg when the patient is not even aware of its existence till felt in the calf? or how is it that Crural Phlebitis can produce pain in the back and hip joint before the patient is aware of any thing being wrong about the crural veins? If we look to the nerves for the source of these pains, we can readily account for them; those large muscular branches which are given off to those large muscles in the calf of the leg, and also in the ham, are irritated at their origin and have become excited, exquisitly sensible, and have produced a tonic contraction of those muscles; the pain always resembles a cramp pain, described as the flesh pulling off from the bones. Says Churchill: "When the disease begins in the pelvis"—and it should always begin there according to Dr. Lee's theory—"the pain speedily extends below pouparts ligament down the thigh, to the ham, calf of the leg and foot." The pain always passes out under pouparts ligament when the lumbar ganglia are affected, but never when confined to the sacral ganglia; it then affects the ham or calf of the leg.

Says Churchill: "Shortly after the commencement, the inguinal region is tumefied, white, and shining. This swelling may be confined to the thigh, or extend down to the heel, and it will very much in amount, occasionally the leg is enormously increased in size."

Says Churchill: "When the pain originates in the back and hips, the nates and vulva become swollen, glassy and tense.—When the disease commences in the calf of the leg, the swelling is first observed there or at the ankles, gradually extending itself up the leg and thigh." The situation of the swelling and pain depend on the portion ot the spinal column affected, whether it be the lumbar or sacral portion, or both—when I speak of lumber or sacral portion, I mean that portion of the chord from which the lumbar and sacral nerves originate.

Churchill, Denman, Burns, Bedford, Megs, and perhaps others, speak of many cases being first attacked in the calf of the leg, and when attacked there, the swelling begins there. How can these symptoms be reconciled with Crural Phlebitis? Why should the swelling commence in the calf of the leg if it is owing to obstruction in the crural veins? Let us see how we are to account for the swelling of the limb—as I believe there always has been some difficulty in accounting for so much swelling in the limb, and yet one of the most usual symptoms of inflammation (redness) is wanting, the limb being even whiter than natural. We have irritability, sensation being extremely acute, and veinous circulation partially arrested. But this obstruction in the veinous circulation is not necessarily owing to the large veins being plugged up, as we will endeavor to show. The swelling is produced, no doubt, by an effusion of Lymph, and not of Serum, into the cellular membrane. Any obstruction in the circulation produces effusion, but it requires an excitement in the nerves, increase of sensibility, or irritability, connected with obstruction in the circulation, to produce an effusion of Lymph, whereas obstruction alone, or even a languid circulation, may produce an effusion of Serum. And this effusion is not Serum, but Lymph, is clearly proven by the limbs not pitting, and in some cases the limb remains permanently enlarged for a long time, which could not be the case if the effusion was Serum.

But to show how the circulation is obstructed without the large veins being plugged up, it will be necessary for us to say a few words in reference to the physiology of the capillary circulation, in which we believe the obstruction exists.

1st. By what force is the capillary, and also the veinous circulation carried on, and where does that force exist? We believe it is in the capillary vessels themselves, and that force to be capillary attraction and chemical affinity. Man would have no more use for a heart than vegetation has, if all his blood-vessels were capillary vessels; the heart can have little influence only on the circulation of the blood through the large ar teries; when it arrives at the capillaries, it is then carried on by capillary attraction, and chemical affinity, as explained by Dr. John W. Draper, and would be as regularly performed in man as in vegetation, were it not that the circulation in man is greatly under the control of the nervous system, either by affecting the caliber of these vessels, or by interfering in some way with assimilation and secretion, thereby preventing the chemical action from taking place, which is necessary to change the blood from arterial to veinous, which must necessarily destroy the force by which the capillary and veinous circulation is carried on.

If the above theory be true, which we believe has been clearly demonstrated, then we have the capillary circulation most effectually arrested, and veinous circulation greatly retarded in the disease before us, by the irritable condition of the diseased nerves, producing complete spasm of the capillary vessels and of muscular fibre, thereby preventing the blood from entering the capillary vessels, showing most conclusivly the cause of the whiteness of the limb. If the crural and illiac veins were closed up—as they must inevitably be in many cases; if adhesive inflammation existed from the first, as stated, the limb would certainly have a livid appearance, instead of shining whitness. Is not this the case in all other cases where there are veinous obstructions? Besides we cannot believe that those large veins can be permanently obstructed without producing much more serious consequences than usually occurs in Phlegmasia Doleus.

We have been thus tedious in giving our views of the cause of the swelling, because there is more difficulty, perhaps, in accounting for the extreme swelling, while the limb remains even whiter than natural, than any other symptom which occurs in the disease. The swollen ridge which is sometimes found in the region of the crural vessels, feeling like a cord, may be owing to an effusion of Lymph into the cellular substance which surrounds those vessels.

Again, Dr. Megs terms this disease "Crural Phlebitis," and describes it as follows: "Now, gentlemen, see a limb supplied by a great femoral artery and propunda, with its anterior and posterior tibials, and all the other branches; imagine these arteries healthy, strong, perfect in structure and volume; and see a current of blood driven into them by the contraction of the heart under a powerful excitement of a great fever; and then imagine the femoral vein or saphena obstructed by thickening of its endangium—by quantities of plastic Lymph exuded from the inner wall—by strong, firm coagula of blood," &c.; "for," says he, "the vein which carries the blood from the limb is virtually compressed, as if you had tied it up with a ligature preparatory to bleeding it. The vein is as if you had put the pad of Petit Tourniquet upon it and left the artery perfectly free and unobstructed." He says now, "tell me what is to become of all the blood thrust into the limb by the injection force of an infuriated systemic ventricle, and how is it to get out?" (a question, I think, much easier asked than answered, if the condition of the veins are as above described.) "How is the woman to avoid the great swelling of the imb which you call Phlegmasia Dolens alba, or milk leg, but which you would better call Crural Phlebitis?" True, were we to tie the veins, or apply the pad of a tourniquet to the veins, we should most inevitably have the swelling, but, I ask, would we have the alba? How are we to account for the extreme whiteness of the limb which always occurs when uncomplicated, if the swelling is owing to the large amount of blood injected into the limb—and the veins being obstructed cannot remove it—would not the limb be of a livid color, instead of a shining whiteness? and is not this the case, if we apply a ligature or a tourniquet to the limb, as above stated? Who has not frequently seen this dark livid appearance of the arm, when he has applied a ligature, preparatory to bleeding, when it has remained a few minutes before the vein was opened? Again says Dr. Megs: "The inflammation of the endangium of the Crural vessels dips through the fibrous coat, and causes infiltration into the common sheath, producing pain, essentially a nuralgia, by pressure on the Crural nerve." Does this account for the pain? or can we reasonably account for it in this way? Is there not frequently violent pain before there is any swelling at all? and, in a majority of cases, is not the pain first felt in the calf of the leg? and does not Dr. Megs himself say, "that in many of the cases he had met with, his first detection of the existence of the disease was made in consequence of complaints as to pain in the calf of the leg?" He says: "When a woman who has been confined, or who is pregnant, tells me she has pain in the calf of her leg, I put a thumb upon the spine of the tibia, and the fingers upon the calf, and then suddenly compress the gastrocnemii and soleas against the bone. If the woman shrinks from the pressure, and makes an outcry, I next examine the groin. If I find the swollen ridge of the theca, or sheath of the vessels, I know my patient labors under Crural

Phlebitis." Again says he: "Direct the woman to lie on her back, with both of the knees up in the bed, until the tibiæ become nearly vertical. You now take hold of the calf of the leg lightly, from behind, and endeavor to shake it from side to side. You will find you can't shake it; for the whole mass seems attached to the bone, or packed against it. If you shake the other calf it will be perfectly flabby and moveable in your hand. The result of this comparison will settle your diagnostic."

So we see Dr. Megs makes the condition of the calf of the leg a diagnostic symptom of Phlegmasia Dolens alba; and yet he calls it Crural Phlebitis. How Crural Phlebitis could produce spasmodic action of the muscles in the calf of the leg, we must acknowledge we have ever been unable to comprehend. In all cases which we have seen, whether the pain was situated in the calf of the leg or the thigh, it has always been described to us, by the patient, as of a nuralgic or spasmodic character, in the commencement of the disease.

Again: What is the treatment which Dr. Megs relies on for the cure of this disease which he calls Crural Phebitis, which, perhaps, is as good treatment as can be adopted? He first speaks of applying leeches to the ridge which he finds on the inside of the thigh, and also recommends Dr. Physick's treat_ ment to apply a blister to the ridge. But he says the limb must be stuped. Procure an old flannel petticoat—where there is a woman there is a petticoat—dip the whole of it into a large basin filled with equal quantities of vinegar and water—the liquor must be very hot. Let the petticoat be wrung out as hard as four hands can possibly wring it, and with it, let the whole member be enveloped, and a blanket rolled over the whole stupe, keeping up its temperature and moisture for a long time. My custom is to keep up the stuping for five con secutive hours; after which, I cause the member to be gently bathed with a mixture of sweet oil and laudanum, carefully wrapping it up in fine flannel; and so I alternate the stuping and the innuction with the oil and laudanum, until the swelling has abated, or until I can shake the calf of the leg;" or, he should have said, relieved the spasm of those muscles.

The above treatment is as good as any that can be adopted, as I have already stated; yet it is certainly a treatment much better calculated to relieve spasm or nuralgia, or an irritation located in the nervous system, than to relieve Phlebitis. Then, to recapitulate: The type of fever; the irritable and depressed state of the system; the character of the pain; the increased sensibility and loss of motion in the limb; the tonic contraction of the muscles; the whiteness of the limb; the swelling always observing a median line; the labium of the diseased side participating in the swelling, but never extending to the other labium, unless the other side is affected; the favorable termination of the disease, all indicate a disease located in the nervous tissue.

We have satisfied ourselves, sometime since, that irritation located in the uterus—not only in the lying-in woman, but in others—is capable of producing disease through the medium of the spinal column, in distant parts, not only functional, but organic, however much we have failed to satisfy others.

We further believe, that those cases which have occurred after aggravated cases of typhoid fever, which so much resembles milk leg, as stated by Dr. Tweedie and others, occur in a similar manner, only the primary disease, or irritation, is located in the mucous membrane of the bowels, instead of the uterus, and is much less likely to be transmitted to the spine than when located in the uterus, owing to the uterus being much more intimately connected with the spine than the mucous membrane of the bowels is.

